# Matrix-Induced Sugaring-Out: A Simple and Rapid Sample Preparation Method for the Determination of Neonicotinoid Pesticides in Honey

**DOI:** 10.3390/molecules24152761

**Published:** 2019-07-30

**Authors:** Wenbin Chen, Siyuan Wu, Jianing Zhang, Fengjie Yu, Jianbo Hou, Xiaoqing Miao, Xijuan Tu

**Affiliations:** 1 College of Bee Science, Fujian Agriculture and Forestry University, Fuzhou 350002, China; 2 College of Food Science, Fujian Agriculture and Forestry University, Fuzhou 350002, China; 3 Zhejiang Academy of Science and Technology for Inspection and Quarantine, Hangzhou 310016, China

**Keywords:** sample preparation, sugaring-out, liquid-liquid extraction, neonicotinoid, honey, HPLC

## Abstract

In the present work, we developed a simple and rapid sample preparation method for the determination of neonicotinoid pesticides in honey based on the matrix-induced sugaring-out. Since there is a high concentration of sugars in the honey matrix, the honey samples were mixed directly with acetonitrile (ACN)-water mixture to trigger the phase separation. Analytes were extracted into the upper ACN phase without additional phase separation agents and injected into the HPLC system for the analysis. Parameters of this matrix-induced sugaring-out method were systematically investigated. The optimal protocol involves 2 g honey mixed with 4 mL ACN-water mixture (*v*/*v*, 60:40). In addition, this simple sample preparation method was compared with two other ACN-water-based homogenous liquid-liquid extraction methods, including salting-out assisted liquid-liquid extraction and subzero-temperature assisted liquid-liquid extraction. The present method was fully validated, the obtained limits of detection (LODs) and limits of quantification (LOQs) were from 21 to 27 and 70 to 90 μg/kg, respectively. Average recoveries at three spiked levels were in the range of 91.49% to 97.73%. Precision expressed as relative standard deviations (RSDs) in the inter-day and intra-day analysis were all lower than 5%. Finally, the developed method was applied for the analysis of eight honey samples, results showed that none of the target neonicotinoid residues were detected.

## 1. Introduction

Honey is a natural and healthy sweetener, which has been widely used for both nutritional and medical purposes [1]. Since the production of honey is inextricably linked with environment and beekeeping practices, honey could be contaminated from a variety of sources [2]. Pesticide contaminants from crops and beehives have gained great attention because of their potential to harm the health of human and honeybee [3]. For decades, neonicotinoid pesticides are extensively used in agriculture due to their distinct characters, such as high efficacy, long-lasting effect, and superb plant-systemic activity [4]. In addition, neonicotinoid pesticides are also widely applied in seed dressings [4]. These can result in the presence of neonicotinoid residues in plant pollen or nectar. As a result, exposure of honeybee and beehive products to neonicotinoid pesticides are envisaged [5].

In recent years, a few publications have reported analytical methods for the determination of neonicotinoid pesticides in honey. Considering the complex matrixes of honey samples, sample preparation procedures needed to be proposed prior to the chromatography separation [6]. For example, different variations of the QuEChERS (quick, easy, cheap, effective, rugged and safe) method have been developed for the extraction and cleanup of neonicotinoid pesticides in honey [7,8,9,10]. Solvent extraction followed by the salting out and C18 solid phase extraction (SPE) cleanup was applied to receive a high recovery of neonicotinoid pesticides [11]. In addition, SPE cartridges with different sorbents were also used for the analysis of neonicotinoids and their metabolites in honey [12,13,14,15]. Dispersive liquid-liquid microextraction (DLLME) was developed by Jovanov et al. [16], and the high enrichment factor of DLLME is helpful to improve the sensitivity of the proposed method [16,17,18]. Additionally, the combination of SPE with DLLME was firstly reported by Campillo et al. to reduce interferences and efficiently preconcentrate neonicotinoid pesticides in honey samples [19]. Recently, Song et al. reported an anion exchanger-disposable pipette extraction (DPX) method for removing sugars from the honey matrix and for the determination of neonicotinoid residues in honey [20]. Although different sample preparation methods have been developed for the analysis of neonicotinoid pesticides in honey, long preparation time and sophisticated procedures are required. In the present work, a novel simple and rapid sample preparation method, which employs matrix-induced sugaring-out is developed for the determination of neonicotinoid pesticides in honey.

Sugaring-out is a phase separation method that by introducing monomeric sugars or disaccharides into acetonitrile (ACN) aqueous solution, ACN can be separated from the water to form a new phase [21]. This novel two-phase system shows the advantages of fast phase separation and is environmentally friendly and has found applications in protein purification [22], natural products extraction [23,24], and bioanalysis [25]. Recently, we reported a sugaring-out assisted liquid-liquid extraction (SULLE) method for the partition of 10-hydroxy-2-decenoic acid (10-HDA) in royal jelly [24] and multiple phenolic compounds in propolis [26] and a SULLE-based sample preparation method for the determination of bisphenol residues in royal jelly [27]. Honey is a natural sweetener composed of ca. 70% (*w*/*w*) monosaccharides [1], thus sugaring-out phenomenon has the potential to be observed when honey is mixed with the ACN-H_2_O solution. The sugaring-out in the honey sample was first reported by Tsai et al. for the determination of sulfonamides [28]. More recently, an application in the quantification of 17 phenolic compounds was developed by Zhu et al. [29]. In this paper, the matrix-induced sugaring-out, which took advantage of the phase separation of ACN-H_2_O mixture induced by honey matrix, was applied for the sample preparation of neonicotinoid pesticides in honey. Parameters of the proposed method were systematically investigated, and the developed method was fully validated and demonstrated to be simple and efficient.

## 2. Results and Discussion

### 2.1. Extraction Solvent

Sugaring-out assisted liquid-liquid extraction (SULLE) is a novel sample preparation method in which sugar is used to trigger phase separation [21,25,27]. ACN [21] and ethanol [30] have been reported to be applied as extraction solvents for sugaring-out. In the honey matrix, ethanol was found to be hardly separated from the ethanol-water mixture. ACN was the most used extraction solvent in sugaring-out studies, which has advantages of rapid phase separation, excellent compatibility with liquid chromatography, and high recovery for polar compounds [26]. Thus, ACN was used in the present study.

### 2.2. Initial Content of Acetonitrile and Sample Amount of Honey

In SULLE, sugars, typically glucose, fructose, or sucrose, were used as a phase separation agent to force out ACN from the ACN-water mixture [21]. In the present work, due to the high concentration of sugars (ca. 70% *w*/*w* of glucose and fructose) in honey, phase separation was quickly observed when the ACN-water solution was mixed with a certain amount of the honey sample. We aim to demonstrate that this matrix-induced phase separation phenomenon can be applied for the pretreatment of the honey sample for the determination of three typical neonicotinoid residues (imidacloprid, acetamiprid, and thiacloprid). Our previous result on the partition of multiple phenolic compounds in ACN-water-based SULLE by glucose has demonstrated that the volume of the separated ACN phase was dependent on the concentration of sugar and the initial content of ACN in the ACN-water mixture [26]. Specifically, increasing the concentration of sugar and the initial content of ACN would both dramatically increase the volume of the upper ACN phase. Thus, for the design of matrix-induced sugaring-out in the honey sample, the initial content of ACN and the sample amount of honey would be two important parameters which significantly influence the phase separation process.

**Figure 1:**
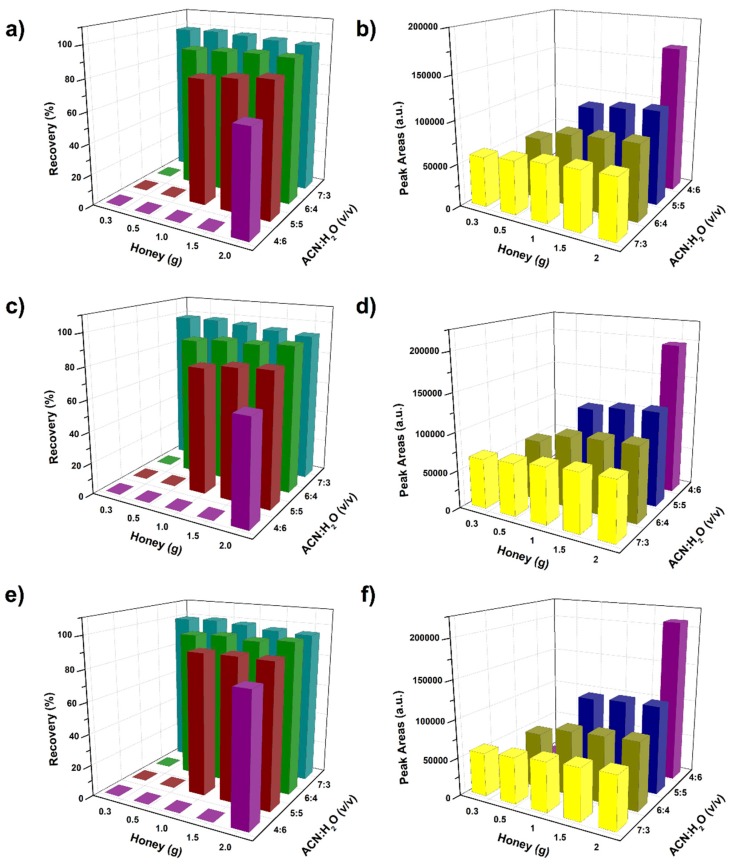
Recovery (**a**,**c**,**e**) and peak areas (**b**,**d**,**f**) of neonicotinoid pesticides under different amounts of honey and different concentration of ACN in ACN-H_2_O mixture. Imidacloprid: (**a**,**b**); acetamiprid: (**c**,**d**); thiacloprid: (**e**,**f**). All the experiments were performed in triplicate, and the mean values were presented.

Effects of ACN content in the ACN-H_2_O mixture and sample amount of honey on the calculated recovery are shown in Figure 1. As shown in Figure 1a,c,e, higher amounts of the honey sample was required to trigger the phase separation when the content of ACN was decreased. For instance, the required amount for the phase separation was reduced to 0.3 g when the content of ACN was 70:30 (*v*/*v*, ACN:H_2_O), however, when the content of ACN was 40:60 (*v*/*v*, ACN:H_2_O), the phase separation was only observed under the sample amount of 2 g. More importantly, increasing the content of ACN dramatically improved the recovery of the investigated neonicotinoid pesticides. For example, recovery of imidacloprid increased from 65.82% to 92.04% as the content of ACN increased from 40:60 (*v*/*v*, ACN:H_2_O) to 60:40 (*v*/*v*, ACN:H_2_O). Then the value of recovery further increased to 95.23% when the content of ACN reached 70:30 (*v*/*v*, ACN:H_2_O). This observation was consistent with our previous reports on the sugaring-out extraction of 10-HDA [24] and bisphenol contaminants [27] in the royal jelly sample, in which the improvement of extraction yields can be attributed to the significant increase of phase ratio when the content of ACN increased.

In addition, increasing the sample amount of honey also exhibited a positive effect on the improvement of recovery. As shown in Figure 1a,c,e, recovery of neonicotinoid pesticides increased as more honey sample was used at the concentration of 50:50 (*v*/*v*, ACN:H_2_O). However, the increment of recovery was reduced at the concentration of 60:40 (*v*/*v*, ACN:H_2_O). Additionally, at the concentration of 70:30 (*v*/*v*, ACN:H_2_O), recoveries were varied slightly when the sample amount of honey increased from 0.3 g to 2 g. These results suggested that extraction yields were increased as the sample amount of honey increased, then reached a plateau when the concentration of honey was above the critical concentration. The high recovery values under the high concentration of sugars were also observed in sugaring-out extraction of protein and organic compounds by glucose [22,24,26,27]. This indicates that we can use a larger amount of honey sample under the high concentration of ACN to improve the sensitivity of the proposed matrix-induced sugaring-out procedure. It is valuable to notice that as the sample amount of honey was above 2.5 g, the insoluble matter was obviously observed. Thus, 2 g would be the suitable sample size in this sugaring-out extraction.

In addition to the recovery results, the concentration of ACN and the sample amount of honey would also influence the concentration of target compounds in the upper phase. Effects of sample amount and ACN content on the signal response are shown in Figure 1b,d,f. Results indicated that peak areas of target compounds were reduced as the ACN concentration increased. When the content of ACN was 40:60 (*v*/*v*, ACN:H_2_O), the least volume of upper phase was obtained, which made the concentration of target compounds and the resulted signal response both at their highest value. However, as discussed above, the recovery of all three pesticides was at the lowest value, thus this condition was not suitable for the sample preparation. ACN content of 60:40 (*v*/*v*, ACN:H_2_O) and 70:30 (*v*/*v*, ACN:H_2_O) are two conditions with recoveries larger than 90%. As shown in Figure 1b, the signal response at the concentration of 60:40 (*v*/*v*, ACN:H_2_O) was much larger than that of 70:30 (*v*/*v*, ACN:H_2_O). Therefore, considering the high value of both recovery and signal response, ACN content of 60:40 (*v*/*v*, ACN:H_2_O) was selected as the suitable condition for the sugaring-out procedure. At this concentration, increasing the amount of honey sample will help to improve both the signal response and the detection sensitivity. The composition of sugar in different types of honey was about 65%–80% (*w*/*w*) [31], which means that the varying sugar contents would be less than 15% (*w*/*w*) in different honey samples. As observed in Figure 1, the value of recovery and peak area reached a plateau when the amount of honey was above 1.5 g. Thus, the selected sample amount of 2.0 g would make the effect of the varying sugar contents on recovery and peak areas become negligible. Therefore, ACN concentration of 60:40 (*v*/*v*, ACN:H_2_O), and a sample amount of 2 g were selected as the optimal preparation protocol.

### 2.3. Comparison with Salting-Out and Subzero-Temperature Assisted Liquid-liquid Extraction

**Figure 2:**
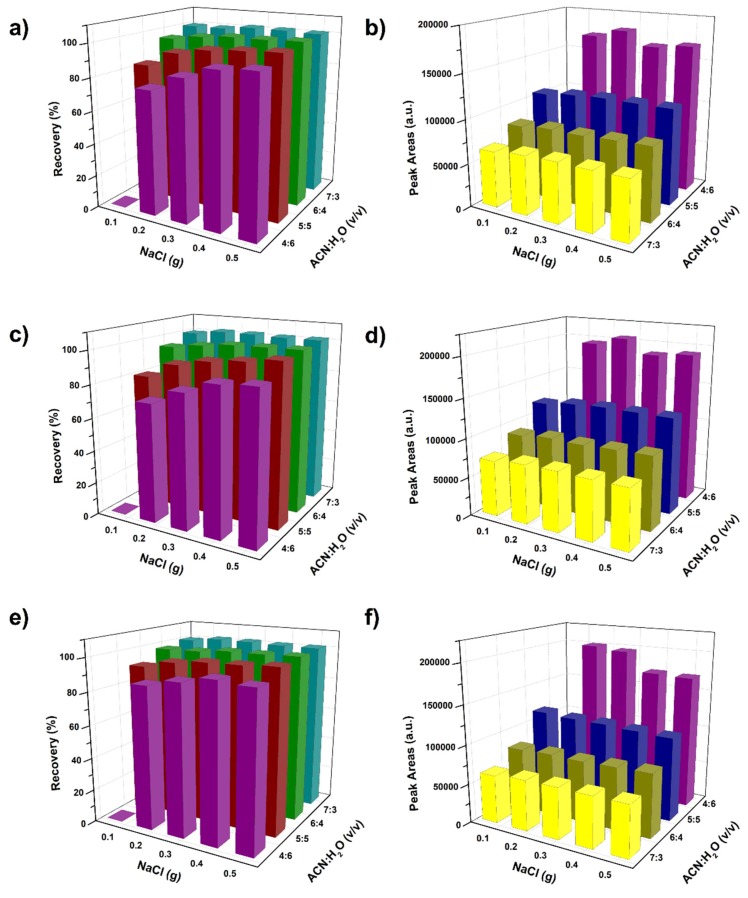
Recovery (**a**,**c**,**e)** and peak areas (**b**,**d**,**f**) of neonicotinoid pesticides under salting out assisted liquid-liquid extraction. Imidacloprid: (**a**,**b**); acetamiprid: (**c**,**d**); thiacloprid: (**e**,**f**). All the experiments were performed in triplicate, and the mean values were presented.

The proposed method was compared with another two ACN-water-based homogenous liquid-liquid extraction methods, salting-out assisted liquid-liquid extraction (SALLE) [32,33] and subzero-temperature assisted liquid-liquid extraction (STLLE) [34]. In the SALLE, different amounts of NaCl were added into the ACN-H_2_O mixture to trigger the phase separation. The recovery and signal response in NaCl-based SALLE are shown in Figure 2. Compared with the sugaring-out extraction, the calculated recovery was slightly higher in SALLE, this may have resulted from the higher phase ratio in SALLE than in the sugaring-out extraction under the same ACN concentration [24]. However, the higher phase ratio would lead to a larger volume of the upper phase and the resulted lower signal response compared to sugaring-out (Figure 1b). Another drawback of SALLE is that the suitable sample amount (0.2 g) was much lower than that in sugaring-out (2 g), which would make the detection limit of SALLE higher than that of sugaring-out. In addition, the additional phase separation agent (salts) were required in SALLE, while no additional phase separation agent was used in the proposed sugaring-out method. This was another advantage of matrix-induced sugaring-out over SALLE.

**Figure 3:**
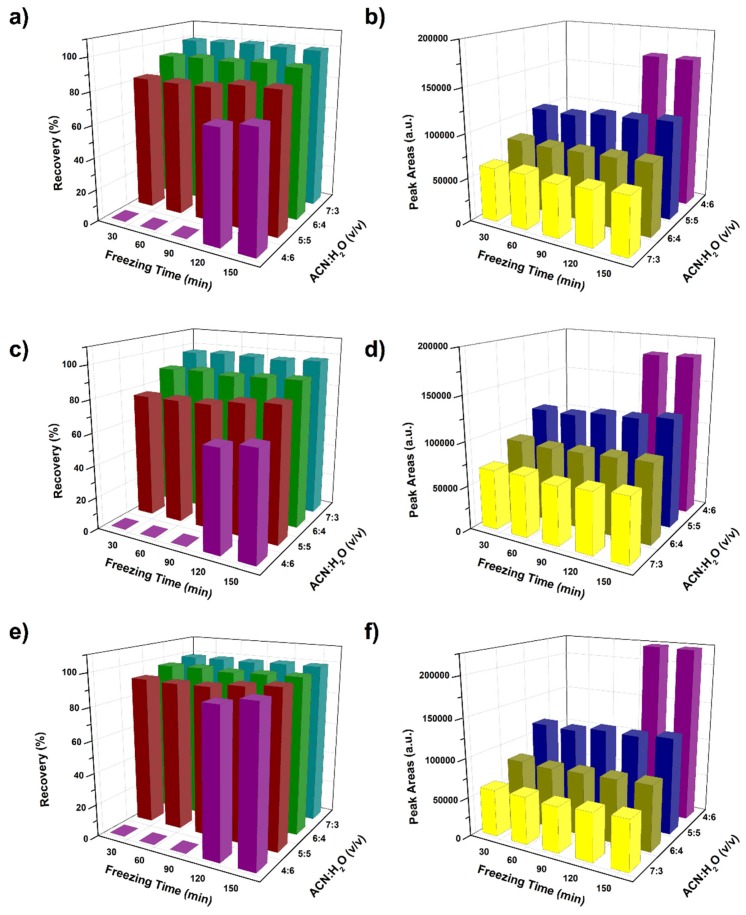
Recovery (**a**,**c**,**e**) and peak areas (**b**,**d**,**f**) of neonicotinoid pesticides under subzero-temperature assisted liquid-liquid extraction. Imidacloprid: (**a**,**b**); acetamiprid: (**c**,**d**); thiacloprid: (**e**,**f**). All the experiments were performed in triplicate, and the mean values were presented.

Subzero-temperature assisted liquid-liquid extraction (STLLE) was another simple homogenous liquid-liquid extraction method, which used the low temperature to make ACN separate from the ACN-H_2_O mixture [34]. In STLLE, the extraction mixture was freezed under −20 °C for different times. The recovery and signal response is shown in Figure 3. Although the calculated recovery in STLLE was slightly higher than that in sugaring-out, the signal response of the final extract in STLLE was much lower than that in sugaring-out. The advantage of STLLE is the elimination of additional phase separation agents, this is also the merit of matrix-induced sugaring-out. Similar to the SALLE, the suitable sample amount in STLLE was 0.2 g, which was much lower than that in sugaring-out. Thus, the matrix-induced sugaring-out method also showed the advantage of higher detection sensitivity compared with STLLE.

### 2.4. HPLC Analysis

**Figure 4:**
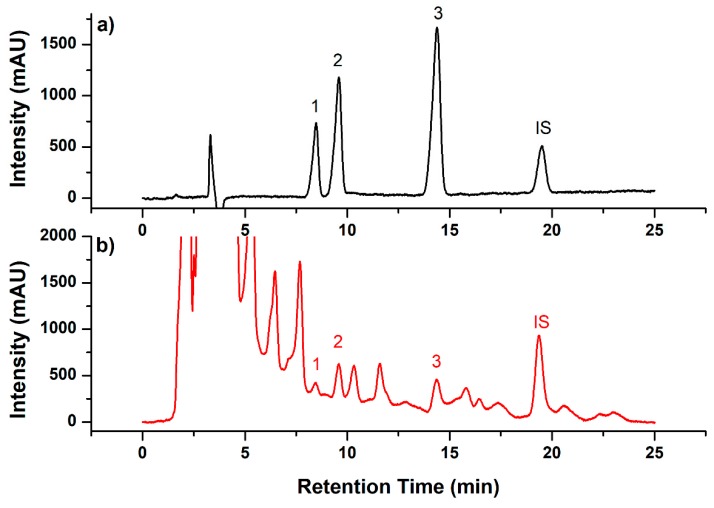
Representative chromatography (λ = 245 nm) of standards (**a**) and spiked chaste honey sample (**b**). Peak 1: Imidacloprid, peak 2: Acetamiprid, peak 3: Thiacloprid, peak IS: Internal standard. The spiked concentration was imidacloprid 70 μg/kg, acetamiprid 70 μg/kg, and thiacloprid 90 μg/kg.

Reversed-phase HPLC was applied for the separation of target neonicotinoid pesticides. The mobile phase was optimized by isocratic elution using mixtures of ACN and water. Different ratios of ACN/H_2_O (*v*/*v*) ranged from 28:72 to 35:65 at a flow rate of 1 mL/min were investigated. When the concentration of ACN was increased, the retention time of target compounds was reduced. However, the resolution between imidacloprid and acetamiprid was also decreased. The optimal chromatographic condition was eluted with 30:70 ACN/H_2_O (*v*/*v*) to obtain good separation in a shorter analysis time (Figure 4). In addition, the injection volumes from 5 to 30 μL were also estimated. Results indicated that 20 μL could be used, as it provided the highest sensitivity without the overlapping of chromatographic peaks. The detection wavelength was optimized by investigating the adsorption profile of target compounds in the diode array detector (DAD) results. A wavelength of 270 nm was used for detecting imidacloprid, and 245 nm was applied for acetamiprid and thiacloprid to obtain high sensitivity.

### 2.5. Analytical Performance

The linear ranges were constructed with seven levels of concentration ranging from 0.05 to 3 μg/mL. Correlation coefficients were all better than 0.9995 in all cases. The limits of detection (LODs, S/N = 3) and limits of quantification (LOQs) for imidacloprid, acetamiprid, and thiacloprid in honey are shown in Table 1. LODs ranged from 21 to 27 μg/kg, and LOQs were between 70 and 90 μg/kg. Accuracy and precision were investigated in spiked honey samples with the addition of three concentration levels (1 × LOQ, 5 × LOQ, and 10 × LOQ), which were also shown in Table 1. The calculated mean recoveries were between 93.78%–97.48%, 91.49%–97.73%, and 91.84%–96.65% for imidacloprid, acetamiprid, and thiacloprid, respectively. The intra-day and inter-day precision were all less than 5%. These results all fulfilled the requirements of the Association of Official Analytical Chemist (AOAC) [35]. The applicability of the developed method was evaluated in 8 honey samples, including acacia, loquat, linden, schefflera, eurya, and codonopsis. The retention time and the spectrum recorded by DAD detector were used for the peak identification. Among these samples, none of the target neonicotinoid pesticides were detected (Figure S1).

In addition, the present method was compared with the reported methods including QuEChERS, SPE, DLLME, and DPX (Table S1). As shown in Table S1, the protocol of the present method was much simpler than the reported methods. Since the phase separation was induced by the honey matrix itself, additional phase separation agents were eliminated in the present method. This can provide important advantages in reducing the consumption of chemicals, simplifying the preparation procedure, and saving time and labor. In addition, we also noticed that the sensitivity of the present method was lower than the compared methods. However, the LOD value was enough for the safety identification according to the regular limit (imidacloprid and acetamiprid, 0.05 mg/kg; thiacloprid, 0.2 mg/kg) [36]. The sensitivity and the number of target neonicotinoid pesticides could be improved by combining this matrix-induced sugaring-out method with more sensitive and selective mass spectrometry detection. These studies are under way.

## 3. Materials and Methods

### 3.1. Materials

ACN (HPLC grade) was supplied by Merck (Darmstadt, Germany). Standards of imidacloprid, acetamiprid, thiacloprid, and ethyl 6-chloropyridine-2-carboxylate (internal standard, IS) were purchased from Aladdin (Shanghai, China). NaCl was obtained from Sinopharm Chemical Reagent Co., Ltd. (Shanghai, China). The stock solutions of standards were prepared by dissolving standards in ACN at a concentration of 1 mg/mL. Working solutions of standards were prepared by further dilution with ACN. All standards solutions were stored at 4 °C until used. Chaste honey collected from Hubei, China was used for method optimization and validation. A total of 8 honey samples used for the survey were obtained from different suppliers. These samples were acacia, loquat, linden, schefflera, eurya (2 samples), and codonopsis (2 samples).

### 3.2. Matrix-Induced Sugaring-Out

Different amounts of honey samples (0.3, 0.5, 1.0, 1.5, and 2.0 g) were spiked with 20 μL standard solutions (100 μg/mL). Then 20 μL IS solution (400 μg/mL) was added into the sample. The spiked sample was mixed with 4 mL ACN-H_2_O solution with concentration (*v*/*v*) ranging from 40/60 to 70/30. After vortex for 1 min, the mixed solution was centrifuged at 6000 rpm for 5 min to obtain a clear phase separation. The upper phase was collected and passed through a 0.22 μm membrane filter, then analyzed by HPLC.

### 3.3. Salting-Out Assisted Liquid-liquid Extraction

Honey samples (0.2 g) were spiked with 20 μL standards solutions (100 μg/mL). Then 20 μL IS solution (400 μg/mL) was added into the sample. The spiked sample was mixed with 4 mL ACN-H_2_O solution with concentration (*v*/*v*) ranging from 40/60 to 70/30. After vortex for 10 s, NaCl (0.1, 0.2, 0.3, 0.4, 0.5 g) was added into the mixture, then vortexed for 1 min. The mixed solution was centrifuged at 6000 rpm for 5 min to obtain a clear phase separation. The upper phase was collected and passed through a 0.22 μm membrane filter, then analyzed by HPLC.

### 3.4. Subzero-Temperature Assisted Liquid-liquid Extraction

Honey samples (0.2 g) were spiked with 20 μL standard solutions (100 μg/mL). Then 20 μL IS solution (400 μg/mL) was added into the sample. The spiked sample was mixed with 4 mL ACN-H_2_O solution with concentration (*v*/*v*) ranging from 40/60 to 70/30. After vortex for 1 min, the mixed solution was cooled at −20 °C for different times (30, 60, 90, 120, 150 min) to trigger phase separation. The upper phase was collected and passed through a 0.22 μm membrane filter, then analyzed by HPLC.

### 3.5. HPLC Analysis

The HPLC system (Shimadzu) composed of LC-20AT pumps, SIL-20AC autosampler, CTO-20AC column oven, and SPD-M20A photodiode array detector. A WondaSil (Shimadzu-GL) C18 column (5 μm, 4.6 × 250 mm) was used for the separation. The mobile phase consisted of 30% ACN and 70% water. The flow rate was 1 mL/min, the injection volume was 20 μL, the column temperature was 22 °C, and the analysis time was 25 min. The detection wavelength was set at 270 nm for imidacloprid and 245 nm for both acetamiprid and thiacloprid.

### 3.6. Method Validation

Calibration curves with seven concentration levels were prepared by standard solutions containing imidacloprid (0.05, 0.1, 0.2, 0.5, 1, 2, and 3 μg/mL), acetamiprid (0.05, 0.1, 0.25, 0.5, 1.0, 1.5, and 3 μg/mL), thiacloprid (0.05, 0.1, 0.2, 0.5, 1, 2, and 3 μg/mL), and IS (2 μg/mL). The ratio of peak area (analyte/IS) vs. the ratio of weight (analyte/IS) was used to construct the analytical curves. The y-intercept was set to 0 and a linear fit was performed. Limits of detection (LODs) and limits of quantification (LOQs) were determined in spiked honey samples as 3 and 10 times the signal-to-noise (S/N) ratio, respectively. Accuracy and precision were evaluated by analyzing the samples spiked at 3 concentration levels (1 × LOQ, 5 × LOQ, and 10 × LOQ). Accuracy was expressed as the percentage of recovery, and precision was measured as a relative standard deviation to the mean recovery of both intra-day (n = 6) and inter-day (n = 18, three consecutive days) analysis.

## 4. Conclusions

In summary, a simple and rapid sample preparation method based on matrix-induced sugaring-out was developed for the determination of three neonicotinoid pesticides in honey samples. The parameters of this novel method, including the effects of the sample amount and the initial ACN concentration on the recoveries and response signal, were systematically investigated. The method was fully validated and demonstrated to be simple, fast, low cost, and highly sensitive. The proposed matrix-induced sugaring-out liquid-liquid extraction method might find applications in the pretreatment of other food samples with a high concentration of sugars.

## Figures and Tables

**Table 1:** Linearity, sensitivity, accuracy, and precision of the present method.

Analytes	Linear Equation	r^2^	LOD (μg/kg)	LOQ (μg/kg)	Accuracy and Precision				
					Spiked Content (μg/kg)	Mean Recovery in Intra-day ± SD (n = 6, %)	Intra-day RSD (n = 6, %)	Mean Recovery in Inter-day ± SD (n = 18, %)	Inter-day RSD (n = 18, %)
imidacloprid	y = 0.280x	0.9999	21	70	70	96.20 ± 2.18	2.27	97.48 ± 3.42	3.51
					350	94.75 ± 1.24	1.31	94.65 ± 1.36	1.44
					700	93.78 ± 1.21	1.29	93.87 ± 1.23	1.31
acetamiprid	y = 0.054x	0.9998	21	70	70	97.73 ± 4.27	4.37	96.99 ± 4.44	4.58
					350	94.85 ± 1.44	1.52	94.83 ± 1.86	1.96
					700	91.49 ± 2.31	2.52	92.24 ± 1.71	1.85
thiacloprid	y = 0.063x	0.9995	27	90	90	92.21 ± 2.73	2.96	91.84 ± 2.76	3.01
					450	94.72 ± 1.77	1.87	92.88 ± 2.07	2.23
					900	96.65 ± 2.46	2.55	96.13 ± 1.99	2.07

## Supplementary Materials

The following are available online, Figure S1: Chromatography of standards and investigated honey samples; Table S1: Comparison of the proposed method with reported sample preparation methods for the determination of neonicotinoids in honey.
