# Supplementary material for: Matrix-Induced Sugaring-Out: A Simple and Rapid Sample Preparation Method for the Determination of Neonicotinoid Pesticides in Honey
Source: Molecules. 2019 Jul 30;24(15):2761. doi: 10.3390/molecules24152761 (PMC6695813; doi:10.3390/molecules24152761)
Supplement: Supplementary file 1 [file molecules-24-02761-s001.zip › molecules-555999-SI.pdf]

molecules

Supplementary material

Matrix-induced sugaring-out: a simple and rapid sample preparation method for the determination of neonicotinoid pesticides in honey

Wenbin Chen [1,*], Siyuan Wu [2], Jianing Zhang [2], Fengjie Yu [2], Jianbo Hou [3], Xiaoqing Miao [1,2], and Xijuan Tu [1,*]

[1] College of Bee Science, Fujian Agriculture and Forestry University, Fuzhou 350002, China.
[2] College of Food Science, Fujian Agriculture and Forestry University, Fuzhou 350002, China.
[3] Zhejiang Academy of Science and Technology for Inspection and Quarantine, Hangzhou 310016, China.

* Correspondence: wbchen@fafu.edu.cn (W.B. Chen), xjtu@fafu.edu.cn (X.J. Tu).

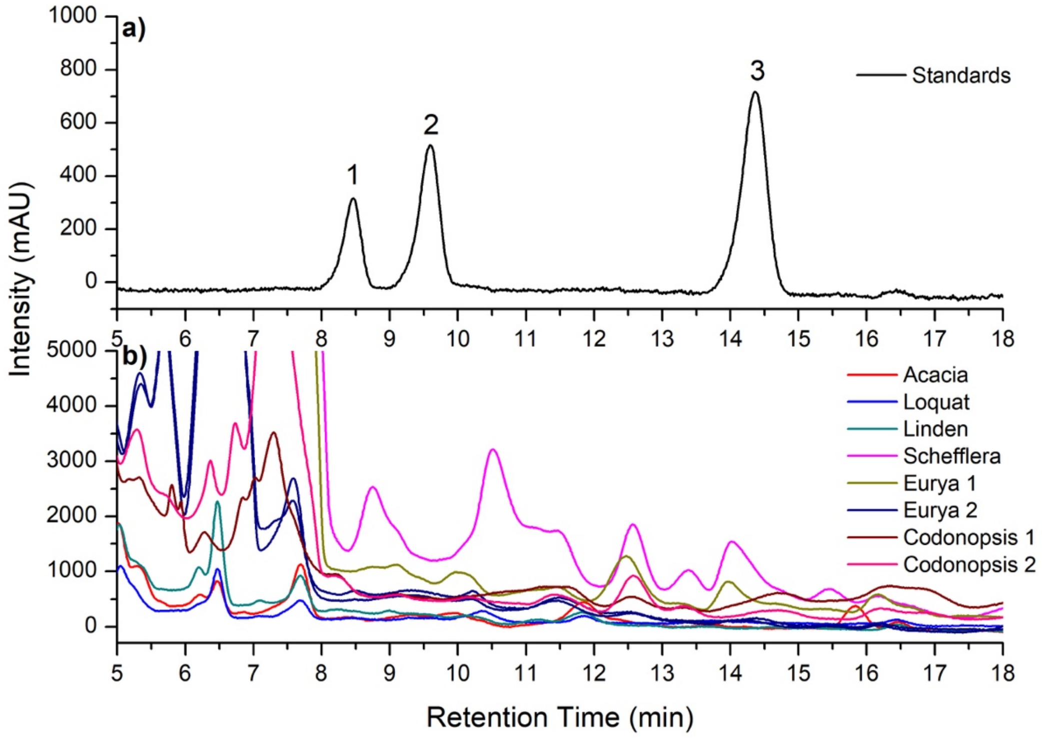

Figure S1. Chromatography (λ=245 nm) of standards (a) and investigated honey samples (b). Peak 1: imidacloprid, peak 2: acetamiprid, peak 3: thiacloprid.

Table S1. Comparison of the proposed method with reported sample preparation methods for the determination of neonicotinoids in honey

Method	Protocol	Detection method	LOD (μg/kg)	Ref.
QuEChERS	Five grams honey were mixed with 10 mL water and 10 mL ACN in a 50 mL centrifuge tube. The mixture was vigorously shaken by hand until a homogenous solution was obtained. After that, 4 g of anhydrous magnesium sulfate, 1 g of sodium chloride, 1 g of trisodium citrate dihydrate, and 0.5 g of disodium hydrogen citrate sesquihydrate was added to the tube. Then the tube was shaken vigorously by hand for 1 min and centrifuge for 5 min. An aliquot of 6 mL of the upper phase was transferred into another tube containing 900 mg of anhydrous magnesium sulfate and 150 mg of PSA. The tube was shaken vigorously by hand for 0.5 min and centrifuge for 5 min. Two milliliter of the supernatant was evaporated to dryness and reconstituted in 1 mL of methanol/water 20:80 (v/v) and for the analysis.	LC-MS/MS	0.6-5	[7]
SPE	Fifteen grams honey were mixed with 12 mL of water and 15 mL of 2% triethylamine (TEA) in ACN with a tissuemizer for 3 min. After that, 6 g of anhydrous magnesium sulfate, 1.5 g of sodium acetate was added to the tube. Then the tube was shaken for 2 min and centrifuge for 5 min. The organic supernatant was transferred into another tube containing 500 mg of anhydrous magnesium sulfate and shaken. Twelve milliliter of the extract was pass through a C18 SPE cartridge preconditioned with 3 mL of 2% TEA in ACN and rinsed with an additional 10 mL of 2% TEA in ACN. The combined eluents were evaporated to dryness and reconstituted in 1 mL of methanol/water 75:25 (v/v) and for the analysis.	LC-MS/MS	0.2	[11]
DLLME	Five milliliter of honey aqueous solution (50.0 g/L), 0.5 mL ACN (dispersive solvent) and 2.0 mL DCM (extraction solvent), were added into the 10 mL round-bottom tube. The extraction tube was shaken for 1 min by vortex and soaked for 10 min in the ultrasonic bath and once more shaken for 1 min by vortex. Then the tube was centrifuged for 5 min. The sediment phase was removed and collected in another tube. The DCM was evaporated and reconstituted in 0.5 mL mobile phase and vortex for 2 min. The final solution was used for the analysis.	HPLC-DAD	1.5-2.5	[16]
DPX	Five grams honey were shaken vigorously with 10 mL ultrapure water for 2 min. After centrifugation, 5.0 mL of the homogenized sample was mixed with 1.0 mL of saturated NaCl. Then 2 mL of this mixture was drawn into the anion exchanger-DPX column with air for mixing, allowed to sit for 30 s, and then discarded. Subsequently, 2.0 mL of 0.1% ammonia was drawn into the anion exchanger-DPX column with air to mix, allowed to sit for 30 s, and discarded. Finally, 1.0 mL ACN and 0.1 mL of 0.1 M HCl were drawn into the anion exchanger-DPX column and mixed. Approximately 1.0 mL of the elution was used for analysis.	LC-MS/MS	0.3-3	[20]
Matrix-induced sugaring-out	Two grams honey were mixed with 4 mL of ACN/H_2O 60:40 (v/v). After vortex for 1 min, the mixed solution was centrifuged at 6000 rpm for 5 min. The upper phase was collected and used for analysis.	HPLC-DAD	21-27	Present method
